# Fracture of the Patella

**Published:** 1896-02

**Authors:** C. O. Weller

**Affiliations:** Austin, Texas


For the Texas Medical Journal.

### FRACTURE OF THE PATELLA.

BY C. O. WELLER, M. D., AUSTIN, TEXAS.

FRACTURE of the patella, comparatively speaking, is not of very frequent occurrence, yet it is sufficiently so to render it necessary that every practitioner should understand its pathology and treatment. The function of this little bone in the normal operations of the knee joint is a very important one, so much so, that when it is interfered with by fracture, progression is practically abolished. The most common fracture, perhaps, is the single and transverse, though it may be longitudinal or stellate. The causes producing it are, muscular action, or injury from a lick, or falling upon the knee. Anatomists tell us that the knee joint is a ginglymus or hinge-joint, and the bones entering into its composition are the condyle of the femur above, the head of the tibia below, and the patella in front. They are held together by quite a number of ligaments, some of which are exterior to, whilst others occupy the interior of the joint. I will not consume your time by describing or even naming all of these, for they are familiar to you, but mention one that is more intimately connected with our subject than the rest, that is the capsular. "This is a very thin but strong fibrous membrane. It is attached to the femur immediately above its articular surface; below to the upper border and sides of the patella, and the margins of the head of the tibia and interarticular cartilages, and is continuous behind with the posterior ligament. It is strengthened by fibrous expansions derived from the fascia lata, from the vasti and crureus muscles, and from the biceps, sartorius and tendon of the semi-membranosus." Within the knee joint is found a very large synovial sac which follows the capsular investment of the joint, but it is not limited by its attachments, for we find it extends above the articular line of the expanded condyles of the femur and forms a cul-de-sac, which passes upward beneath the quadriceps tendon in front of the femur. In connection with lesions affecting the cavity of the knee joint, the attachments of the vastus internus and crureus, constituting, as they do, practically but one muscle, are important. It arises from nearly the entire length of the internal, anterior and external surfaces of the shaft of the femur; its attachment above is limited by its aponeurotic connection with the lower portion of the line which extends from the inner side of the rear of the femur to the linea aspera. It is also attached to the entire length of the latter at its inner side. A transverse partition is thus formed by this muscular plane between the anterior and posterior structures of the thigh for at least three-fourths of that portion of the femur which lies below the level of the hip joint. The lower portion of the anterior surface of this femur bone is separated from the crureus portion of the vastus internus by the intervention of the synovial membrane of the knee joint. Thus it will be seen that the cavity of this joint extends in an upward direction upon the anterior surface of the femur for at least one-fourth of the entire length of the latter, these terminating upon the plane of the attachment of the vastus internus. In case, therefore, of a rupture of this upper recess of the cavity of the knee joint, there would necessarily occur the passage of serum and blood from the latter, and this would pour out upon the muscular plane above referred to, passing thence upward, and, if sufficient in quantity, or forced out of the cavity of the joint by the application of a bandage, it would find its way along this muscular partition, until arrested by the aponeurotic attachment of its upper limit.

Usually the diagnosis of fractured patella is sufficiently easy; the loss of function of the joint, inability to extend the leg, and the gap between the fragments, indicate quite plainly the nature of the trouble. In longitudinal fracture there is usually some obscurity about the diagnosis, for the separation is slight, and the swelling of the joint renders it uncertain. "Hemorrhage between the fragments occurs in all cases, and therefore communicates with the synovial membrane which is interposed between the posterior surface of the patella and the general cavity of the joint; and in cases where the separation is from one-half to one inch and over, it is more than probable that the reflection of the synovial lining from the lower anterior portion of the joint, below the patella, upward and forward to the front of the inter-condyloid notch, is torn, and that whatever of extravasation occurs, is into the general cavity of the joint. More or less effusion in the joint follows in the majority of cases. Not only this, but in some cases the same force that breaks the bone not unfrequently, tears the upper cul-de-sac of the synovial membrane, that is interposed between the quadriceps tendon and the femur, over the lower fourth of that bone, and allows an extravasation of blood and synovia into the muscular plane, limited by the attachments of the vastus internius and crureus."

Having decided our patient has a fractured patella, what are we to do for his relief? Put him to bed, in the dorsal decubitus, keep the affected limb straight, elevate the foot and leg upon pillows, bring the fragments together, and keep them so by appropriate apparatus; much easier said than done. A posterior splint is to be applied, extending from the gluteal fold to near the ankle, the fragments drawn together and held in apposition by proper appliances. The posterior splint may be made of gut-tapercha, sole leather, shelve board, or a piece of plank, in the absence of the others, will answer the purpose. To hold the fragments together, some use adhesive straps, others the figure of 8 bandage, some Malgaigne's hooks, some plaster of Paris, and others the dangerous and unjutifiable and unnecessary method of opening the knee joint and wiring the fragments. I say unjustifiable, because statistics demonstrate that it is fraught with danger not only to the limb, but also to the life of the patient, even when performed by skillful hands and with strict regard to the principles of aseptic and antiseptic surgery. This operation is sometimes successful, but is often followed by osteaorthritis, suppuration of the joint and pyemia, and if the upper cul-de-sac of the synovial sac has been ruptured, an extension of the inflammatory and suppurative process to the structure of the thigh. This being the case, why should it be resorted to when there are safer and equally effective means in bringing about the desired result, viz., the restoration of the fracture of the joint?

In the Annual of the *Universal Medical Sciences*, Vol. II, pages 265 and 266, Von Bergmann, after quoting the statistics of Divernesse, Brunner and Ruhland, says: "Whatever faults may be charged against the unsuccessful surgeons and their antiseptic measures, the fact of frequent failure can not be disregarded, and all the more, because the majority, the very great majority, of transverse fractures recover with relatively good restoration of function, without suture and without operation, even when the union of the fragments is not close and bony."

The statistics published by Valoquires in the *Archives of Medicine*, show that of forty-three recent cases twenty-seven were successes. Seven resulted in loss of mobility; seven were followed by suppuration, in two of which amputation of the thigh was done; there were two deaths, one by pyemia and one by carbolic acid poisoning. Of forty-five old cases twenty-two were successful; in nine there was partial auchylosis; in eleven complete auchylosis (ten after suppuration of the joint), and three died. He meets the assertion that the failures were due to defective antisepsis, by pointing out that some of them occurred in the practice of surgeons who are known to be among the most skillful and careful.

Wyeth, in his book on surgery, says: "The most unjustifiable method of treatment ever introduced in this fracture, is that of opening into the joint and wiring the fragments." He further says, "In 1881, induced by the reported successes after this operation, I wired a fractured patella on the twentieth day after the accident, in the case of a woman twenty years old. The strictest antiseptic precautions were employed, and free drainage was secured. Osteo-arthritis, with destruction of the joint, resulted, and the patient barely escaped with her life, the limb having been amputated in the lower third of the thigh. Another patient, in the hands of a New York surgeon, died as a result of this operation." "If the full histories of all these cases were written, I think few surgeons would have the temerity to repeat the procedure." But I need not quote statistics any further. I do not say there are no circumstances that justify this operation. In compound fracture, it might be both justifiable and necessary. The proposition I offer is this: That in simple fracture of the patella, opening the knee joint and wiring the fragments together is, taken in the light of the history of the operation and of the experience of skillful surgeons, unjustifiable. It is making a compound fracture out of a simple one, with all of its attendant dangers to limb and life; and even should the patient escape without the loss of either, the function of the joint is no better than if he had been treated by simpler methods.

On the 19th day of last June, a man of good muscular development was standing on the sidewalk looking at a procession that was passing. He had his left foot on the ground, and his right upon a box about a foot high, in front of him. Attempting to raise himself upon the box, it slipped, or turned under him, and in the endeavor to prevent a fall, he threw his right leg violently forward in strong extension. He immediately felt a loss of power over his leg, but managed to drag it along until he entered an office near by. At first he felt no pain, only a sensation of numbness, but after a few minutes the pain was quite severe. After the lapse of a short time, he was driven to his home I was sent for, and, on examination of his knee, found a fractured patella, the interval between the fragments leaving no doubt as to the diagnosis. The joint was swollen and painful. He was placed in as comfortable a position as possible, with the limb in extension, the foot and leg on pillows, elevated. I got a piece of sole leather long enough to reach from the gluteal fold to just above the ankle, and wide enough to embrace, when bent, two-thirds of the circumference of the thigh and leg. A notch was cut about three or four inches above and below the center of the knee joint, on each side, to prevent the bandage from slipping. The leather was soaked in warm water until pliant, then well padded with cotton, an extra piece being placed in the popliteal space, to prevent too much extension. The leg was then placed in the splint, the fragments approximated, and a stout bandage carried around the knee in the form of the figure 8. A roller bandage was then applied from the lower extremity of the splint to the upper, and back again, holding it well in place. I will not bore you with a detail of the treatment during confinement to his bed. There was nothing marvellous either in the treatment or its results. Suffice it to say that at this date, about nine months since the accident, the gentleman has a good ligamentous union of the bone, can extend his leg with ease, and flex it nearly to a right angle with the thigh. As a precautionary measure, however, against two much strain on the newly formed ligament, he still wears a short posterior splint, as a flexion check, which he is expected to continue for some time, until the ligamentous union is strong enough to meet all demands upon it. He is very well pleased with the result. I am but moderately pleased with it. I should have been more so, had there been perfect bony union, but I am consoled by the fact that so great a surgeon as the late Prof. Gross says: "The union always takes place by fibrous or ligamentous tissue, and not by osseous." He continues: "In all the examples of this fracture that I have been able to examine, both in the living subjects and in the museums, I have not met with any in which the consolidation was completely osseous."

In conclusion, let me say: let us not be induced by the triumphs of modern aseptic and antiseptic surgery to perform operations that are not really necessary, for the sake of the eclat that may attach thereto. Aseptic and antiseptic methods have their proper uses and application, and should be followed in all operative procedures, but their powers have a limit, and should not be subjected to failure and disrepute by the rashness of the surgeon. I am conscious of not having done the subject of this paper justice, but, as I conceive it, the object of an essay at a medical society is not to exhaustively treat of its subject, but to elicit the experience of the subject and its discussion by the members.

In the preparation of this paper, I must acknowledge my indebtedness to Gray's Anatomy, the Annual of the Universal Medical Sciences, Reference Hand-book of the Medical Sciences, and Gross and Wyeth's Surgery.

